# Nevirapine Increases Sodium/Iodide Symporter-Mediated Radioiodide Uptake by Activation of TSHR/cAMP/CREB/PAX8 Signaling Pathway in Dedifferentiated Thyroid Cancer

**DOI:** 10.3389/fonc.2020.00404

**Published:** 2020-03-31

**Authors:** Hongxia Shang, Junyu Zhao, Jinming Yao, Huanjun Wang, Jianjun Dong, Lin Liao

**Affiliations:** ^1^Department of Endocrinology and Metabology, Shandong Provincial Qianfoshan Hospital, Cheeloo College of Medicine, Shandong University, Ji-nan, China; ^2^Department of Endocrinology and Metabology, The First Affiliated Hospital of Shandong First Medical University, Ji-nan, China; ^3^Department of Endocrinology and Metabology, Qilu Hospital of Shandong University, Cheeloo College of Medicine, Shandong University, Ji-nan, China

**Keywords:** nevirapine, radioiodide uptake, sodium/iodide symporter, TSHR/cAMP/CREB/PAX8 signaling pathway, dedifferentiated thyroid cancer

## Abstract

Nevirapine has been proved to be effective in inducing re-differentiation and suppressing tumor growth in several tumor cells. This study aims to investigate the therapeutic potential of nevirapine in dedifferentiated thyroid cancer (DeTC), which refractory to radioiodine treatment and the underlying mechanisms. The results indicated that nevirapine significantly inhibited the proliferation and increased the expressions of thyroid differentiation-related genes, thyroid stimulating hormone receptor (TSHR), sodium/iodide symporter (NIS), thyroid peroxidase (TPO), and transcriptional factor paired box 8 (PAX8) in dedifferentiated thyroid cancer cells (WRO 82-1 and dFTC-133). Furthermore, nevirapine also enhanced radioiodide uptake significantly both *in vitro* and *in vivo*, and inhibited the growth of xenograft tumors. Nevirapine might improve radioiodine sensitivity via the activation of TSHR/cAMP/CREB/PAX8 signaling pathway. This study demonstrates that nevirapine could be potentially used to improve radioiodine therapeutic efficacy in dedifferentiated thyroid cancer patients.

## Introduction

Thyroid cancer, the most common endocrine malignancy, has increased substantially worldwide (1). Recently, the incidence of thyroid cancer has risen on average 5% per year, and its mortality has also increased on average 0.9% each year in United States (2). Well-differentiated thyroid cancer (WDTC) is a generally slow-growing tumor with a good prognosis, its 20-year overall survival rate is more than 90% via conventional therapy (3, 4). Notably, thyroidectomy and thyroid hormone suppression therapy with or without thyroid cancer remnant and metastasis ablation by radioiodine treatment are widely accepted as routine therapies for WDTC (2). However, during these treatment, around 30% of WDTC may progress to dedifferentiated thyroid cancer (DeTC), a state occupying an intermediate position in both morphology and behavior between WDTC and anaplastic thyroid cancer (ATC) (5, 6). DeTC was characterized by aggressive growth, distant metastasis, recurrence, in particularly, resistance to radioiodide therapy and finally resulted in powerless in DeTC treatment. Consequently, 10-year survival rate of DeTC declines to ~15–20% (7, 8).

The sodium/iodide symporter (SLC5A5, also named as NIS) is a key cytomembrane glycoprotein that mediates active transport of iodide in the thyroid, which plays a central role in the treatment of DeTC (9–11). The mechanisms of resistance to radioiodine are based on decreased expression of NIS, diminished plasma membrane localization of NIS, or both, which are caused by key genetic and epigenetic alterations and dysregulated signaling pathways (6, 11–15). Many of mutations possibly resulting in thyroid cell dedifferentiation, lead to the activation of two major signaling pathways: phosphoinositide 3-kinase (PI3K) and mitogen-activated protein kinase (MAPK) signaling pathways (2, 16, 17). The use of multi-targeted tyrosine kinase inhibitors, such as lenvatinib and sorafenib, or other FDA-approved anti-tumor drugs to treat radioactive iodine-refractory metastatic DTC, might be beneficial to patients in terms of progression free survival (18). However, limited evidence supports improvement in overall survival. Therefore, further studies are needed to explore new agents that can be more effective in the treatment of patients with DeTC.

Endogenous reverse transcriptase (RT) encoded by endogenous retroviruses and retrotransposons, two kinds of genomic repeated elements, is being used as a novel molecular target in cancer therapy. Growing evidence suggests that the endogenous RT is involved in the regulation of cell proliferation and differentiation since it is highly expressed in embryonic, transformed, undifferentiated, and tumor cells, while expressed at low level in differentiated and non-pathological tissues (5, 13, 19–24). Nevirapine, a non-nucleoside reverse transcriptase inhibitor, is originally used in human immunodeficiency virus (HIV) patients and identified as a differentiating and anti-proliferative agent in human carcinomas in recent years (20, 22, 23).

Nevirapine has been proved to be effective in inducing differentiation and suppressing growth in progenitor cells and several tumor cells (22, 23). But, few studies suggest its effects on DeTC or effectiveness of radioiodine treatment. In our previous study, nevirapine upregulated the levels of NIS and TSHR mRNA, however, to our disappointment, it did not improved the radioiodide uptake significantly in anaplastic thyroid cancer cells (25). In this study, the aim is to investigate the therapeutic potential of nevirapine in DeTC, which refractory to radioiodine treatment and the underlying mechanisms.

## Materials and Methods

### Cell Culture and Human Tissues

The WRO 82-1 cell line was purchased from Sigma-Aldrich (Munich, Gemany) and the FTC-133 cell line was purchased from ATCC (Bethesda, MD). The WRO 82-1 cells and the FTC-133 cells were cultured in RPMI 1640 medium (Gibco, USA) and Leibovitz's L-15 Medium (M&C, China) containing 10% fetal bovine serum (FBS, Gibco, USA), 2 mM L-glutamine (Hyclone, USA) and 1% penicillin/streptomycin (Sigma-Aldrich, USA), respectively. Nevirapine (National Institutes for Food and Drug Control, Beijing, China) was dissolved in dimethyl sulfoxide (DMSO, MP Biomedicals, USA) to prepare a 250 mM stock solution and stored at −20°C. As appropriate for the experiments to be performed, the cells were starved in medium containing 2% FBS for 24 h before they were treated with nevirapine. For some experiments, the adenylyl cyclase inhibitor SQ22536 (9-(tetrahydro-2-furyl)adenine, 10 μM, MCE, China) was added 1 h before nevirapine treatment in medium containing 2% FBS. Human thyroid tissues were obtained from fresh surgical samples as controls.

### dFTC-133 Cell Line Establishment

As previously reported (5, 12), the dedifferentiated FTC-133 (dFTC-133) cell line was established by radiation with ^131^I. Briefly, the FTC-133 cells were seeded in 6-well plates incubated with 15 μCi Na^131^I for 3 days and then cultured in activity free medium by graded dilutions for 3 months, afterwards wells with one cell clone were selected and cells were further cultured. Cells with lowest radioiodine uptake were defined as dFTC-133 and their molecules related to iodide-transport were detected. All relevant results are shown in Supplementary Figure 1.

### Cell Proliferation Assay

Cell Counting Kit-8 (CCK-8; Dojindo, Japan) assay was used to assess cell proliferation/viability according to the manufacturer's instructions. After incubation with nevirapine (100, 200, 350, and 500 μM) or the same volume of 0.1% DMSO (as control) for 24, 48, and 72 h, CCK8 reagent was added into each well, and the cells were incubated at 37°C for 3 h. Cell viabilities were measured through absorbance (optical density) by a microplate reader at a wavelength of 450 nm.

### Annexin V-FITC Apoptosis Assay

The cells seeded in six-well plates were treated with 100, 200, 350, and 500 μM nevirapine or the same volume of 0.1% DMSO (as control) for 72 h, then harvested and stained with annexin V-fluorescein isothiocyanate and propidium iodide (NeoBioscience, China) according to the manufacturer's instructions. Finally, the apoptotic rates were obtained by flow cytometry.

### Western Blot

Western blot analysis was performed using standard protocols as described previously (26, 27). All protein extracts were denatured in a boiling water bath (95°C) for 5 min except for NIS, which was heated at 37°C for 30 min. The antibodies and their dilutions used were as follows: rabbit anti-NIS (1:200; Abcam, UK); rabbit anti-CD97 (1:1,000; Abcam, UK); rabbit anti-GAPDH (1:5,000; Proteintech, China); rabbit anti-Na+/K+-ATPase (1:1,000; Abcam, UK); rabbit anti-cAMP (1:1,000; Abcam, UK); rabbit anti-pCREB (Ser133) (1:1,000; Abcam, UK); mouse anti-TSHR (1:1,000; Abcam, UK); peroxidase conjugated goat anti-rabbit IgG (1:10,000; ZSGB-BIO, China); peroxidase conjugated goat anti-mouse IgG (1:10,000; Proteintech, USA). The quantitative analysis of band intensity was performed by ImageJ software.

### Immunofluorescence

The immunofluorescence analysis was preformed according to previous methods (28, 29). WRO 82-1 and dFTC-133 cells were plated onto 12-mm glass coverslips in a 24-well plate at concentrations of 1.0 × 10^5^ and 1.0 × 10^4^ cells per well with 200 μM nevirapine. After incubation for 72 h, samples were washed with PBS three times, fixed with 4% paraformaldehyde for 15 min, then permeabilized by 0.5% Triton X-100 and blocked with 5% BSA for 30 min at room temperature. For immunofluorescence analysis, samples were stained with anti-NIS antibody (1:50; Abcam, UK) overnight at 4°C, and then incubated with Alexa Fluor secondary antibody (1:200; Life Technologies, USA) in the dark for 1 h at room temperature. Nuclei were stained with DAPI (Life Technologies, USA) 5 min, all followed by washing with PBS for 5 min three times. Finally, coverslips were mounted onto glass slides with antifade solution (Solarbio, China). Images were obtained by a confocal fluorescence microscope (Zeiss, Germany) in the same settings for all specimens.

### Lentiviral Transduction

Lentiviruses carrying shRNA targeting human PAX8 lentiviral vectors (GV248) were constructed by GeneChem. The lentiviruses were used to infect WRO 82-1 and dFTC-133 cells in the presence of Polybrene. Forty-eight hours later, the stable clones were selected by puromycin and the expression of PAX8 in the infected cells was verified by RT-PCR and western blot analysis. The shRNA sequences are below. PAX8, 5′-GAC TAA GCA TTG ACT CAC A-3′; the non-targeting control, 5′-TTC TCC GAA CGT GTC ACG T-3′.

### Quantitative RT-PCR

Total cell RNA was extracted using TriZol reagent (TaKaRa, Japan) according to the manufacturer's instructions. Total RNA (5 μg) was converted to synthesize cDNA using the First Strand cDNA Synthesis Kit (TaKaRa, Japan). RT-PCR was performed on an ABI PRISM 7500 Real-time PCR System (Applied biosystems, USA) using the SYBR Green (GenStar, China). A 20 μL volume reaction consisted of 0.4 μL reverse transcription product and 10 nM primer. The primer sequences are as follows: NIS forward 5′-TCC ATG TAT GGC GTG AAC C-3′, reverse 5′-CTT CGA AGA TGT CCA GCA CC-3′; TSHR forward 5′-TCA TTT GAC ATA GCA GAA AC-3′, reverse 5′- TAA TAG TGA CCA AGT TCT GA-3′; PAX8 forward 5′-TAC TCT GGC AAT GCC TAT GG-3′, reverse 5′- TAC AGA TGG TCA AAG GCC G-3′; GAPDH forward 5′-CAG AAC ATC ATC CCT GCC TCT AC-3′, reverse 5′- TTG AAG TCA GAG GAG ACC ACC TG-3′.

### *In vitro* Iodine Uptake Assay

After treatment with nevirapine (100 and 200 μM) or 0.1% DMSO as control in 24-well plates, the cells were washed with ice-cold modified Hanks' balanced salt solution (HBSS) three times for 5 min. Steady-state radioiodide uptake was determined as follows. The cells were incubated with 2 μCi Na^125^I in 5 mM non-radioactive NaI for 30 min at 37°C. The cells were then washed with cold HBSS and lysed with 500 μL formic acid for 20 min. The radioactivity was measured in the cell lysates by a gamma counter (Packard Bioscience, AU). The radioactivity not contributed by NIS-mediated iodide uptake was conducted by parallel experiments with 80 μM of sodium perchlorate, a selective inhibitor for NIS-mediated iodide uptake. The radioactivity was normalized to the number of viable cells at the beginning of the experiment and expressed as cpm per 10^6^ cells.

### Tumor Xenografts and *in vivo* Iodine Uptake Assay

The male 4-week-old nude mice (Vital River, Beijing, China) were fed under specific pathogen-free conditions for 1 week to accommodate the experimental conditions. The nude mice were then inoculated subcutaneously with WRO 82-1 cells (1 × 10^6^/mouse). Ten days after tumor implant, nevirapine (150 mg/kg/day) was administered orally 5 days a week. The tumor volume was measured twice a week and calculated according to the following formula: length × height × width × 0.52. Three weeks after treatment, 10 μCi Na^125^I were injected intraperitoneally. Animals were sacrificed 4, 24, and 48 h after Na^125^I injection. Iodine uptake was measured in a gamma counter (Packard Bioscience, AU), normalized by weight and expressed as a radioactivity ratio of tumor to thyroid (24). All animal experiments were approved by the institutional ethics committee on animal care and experiment of Shandong Provincial Qianfoshan Hospital.

### Immunohistochemistry

The tumors and patient thyroid tissues were fixed in 10% formalin for 1 day. After dehydration and paraffin embedding, the samples were sliced into 4 μM thick sections and mounted on glass slides. Antigen recovery was performed by pressure-cooking the slides in citrate buffer (pH 6.0) twice for 8 min each time after deparaffinization and rehydration. After blocking endogenous peroxidase activity by 3% hydrogen peroxide, the sections were incubated with rabbit anti-NIS antibody (1:25 dilution; Abcam, UK) at 4°C overnight, followed by incubation with anti-rabbit secondary antibody (Servicebio, China) at 37°C for 50 min. NIS staining was detected using a DAB kit (Servicebio, China) whereas cell nuclei were counterstained with hematoxylin.

### Statistical Analysis

The results of quantitative data were expressed as the mean ± standard deviation (SD). Data were analyzed with GraphPad Prism 5. Statistical significance was determined by two tailed Student's *t-*test analysis based on a *P-*value of <0.05.

## Results

### Low Doses of Nevirapine Inhibited Cell Proliferation Without Inducing Apoptosis in Dedifferentiated Thyroid Cancer Cells

**Figure 1:**
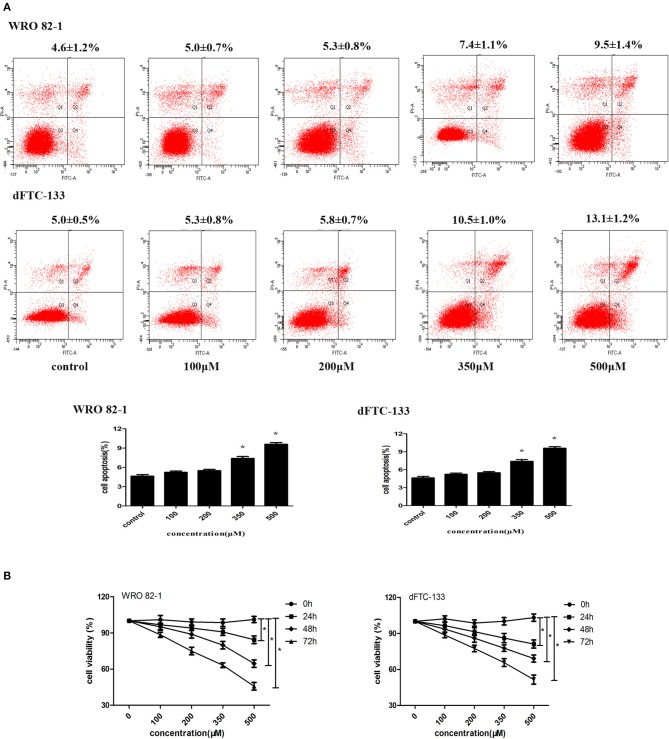
Nevirapine inhibited cell proliferation without inducing apoptosis in dedifferentiated thyroid cancer WRO 82-1 and dFTC-133 cells. **(A)** The apoptotic cells were detected by Annexin V-FITC and PI staining using flow cytometry. The percentage of apoptotic cells is shown as the mean ± SD of three replicate experiments above the panels. **(B)** Effects of nevirapine on cell proliferation was monitored by Cell Counting Kit-8 assay. Data are shown as the mean ± SD of three experiments. *P < 0.05 vs. control.

To analyze the role of nevirapine in regulating apoptosis in WRO 82-1 cells and dFTC-133 cells, an annexin V-fluorescein isothiocyanate and propidium iodide kit was used. After 24 h serum starvation, cells were incubated with nevirapine (100, 200, 350, and 500 μM) for 72 h. We did not observe increase in cell apoptosis in the presence of 100 and 200 μM nevirapine in both cell lines (Figure 1A). By contrast, 7.4 ± 1.1% and 9.5 ± 1.4%, 10.5 ± 1.0% and 13.1 ± 1.2% induction of apoptosis were observed with higher doses of nevirapine (350 and 500 μM), respectively, in WRO 82-1 cells and dFTC-133 cells (*P* < 0.05). The above results indicated that the concentrations of nevirapine (100 and 200 μM) were non-cytotoxic. So, both cells were cultured in the absence and presence of 100 or 200 μM nevirapine.

Cell proliferation was measured by CCK-8 assay. As indicated in Figure 1B, nevirapine suppressed the proliferation of both cell lines in a dose- and time-dependent manner. After incubation with 100 μM nevirapine for 48 h, the viability rates of WRO 82-1 and dFTC-133 cells were 95.1 and 93.6%, respectively (*P* < 0.05). When the concentration of nevirapine reached 200 μM and the cells were incubated with nevirapine for 72 h, the viability rates of WRO 82-1 and dFTC-133 cells were 74.9 and 77.4% (*P* < 0.05).

### Nevirapine Promoted Cell Differentiation in Dedifferentiated Thyroid Cancer Cells

**Figure 2:**
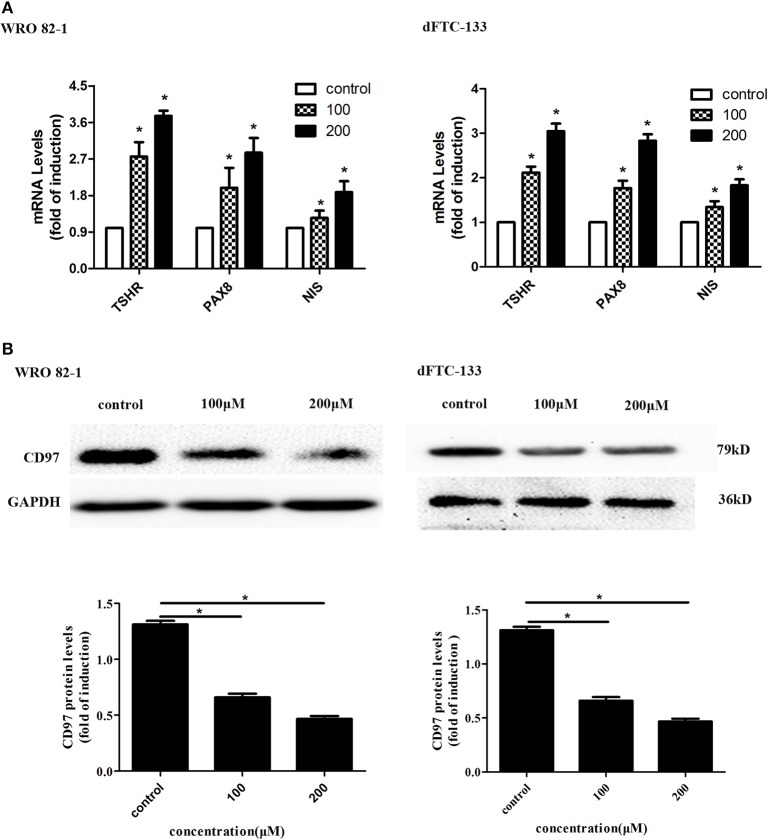
Nevirapine promoted cell differentiation in dedifferentiated thyroid cancer WRO 82-1 and dFTC-133 cells. **(A)** The mRNA levels of thyroid differentiation specific genes (TSHR and NIS) and thyroid transcription factor PAX8 in WRO 82-1 and dFTC-133 cells treated by 200 μM nevirapine for 72 h were detected by RT-PCR. **(B)** The expression of CD97 protein in both cells with 100 and 200 μM nevirapine for 72 h was determined by western blot analysis. GAPDH was used as the loading control. The intensity of CD97 protein bands was quantified. Data are shown as the mean ± SD of three experiments. *P < 0.05 vs. control.

To assess the effect of nevirapine on thyroid differentiation specific genes in WRO 82-1 and dFTC133 cells, the expressions of thyroid differentiation-related genes (TPO, TSHR, and NIS) and thyroid transcription factors (TTF-1, TTF-2, and PAX8) were detected by RT-PCR experiments. The results demonstrated that nevirapine significantly increased the levels of PAX8, TSHR, and NIS mRNA (Figure 2A), but failed to up-regulate the expressions of TTF-1, TTF-2, and TPO mRNA. After 72 h of 200 μM nevirapine treatment, the expressions of NIS mRNA were ~1.9- and 1.8-fold, the mRNA levels of TSHR were about 3.7 times and 3.0 times, and the levels of PAX8 mRNA were ~2.9- and 2.8-fold compared with that in controls in WRO 82-1 and dFTC-133 cells, all had statistical significances (all *P* < 0.05).

CD97, in tumor, is highly correlated with invasion and dedifferentiation (27, 30–32). We analyzed the expression of CD97 in both cells. CD97 expressions were gradually decreased with increased nevirapine concentrations (100 and 200 μM) and cells treated by 200 μM nevirapine had the lowest expression of CD97 (Figure 2B). The levels of CD97 were inbibited by 48.4 and 66.2% in WRO 82-1, and 35.5 and 46.9% in dFTC-133 compared with that of controls (*P* < 0.05). Our data are consistent with the results of previous published studies (30–34).

### Nevirapine Upregulated the Expression of Plasma Membrane-Localized NIS in Dedifferentiated Thyroid Cancer Cells

**Figure 3:**
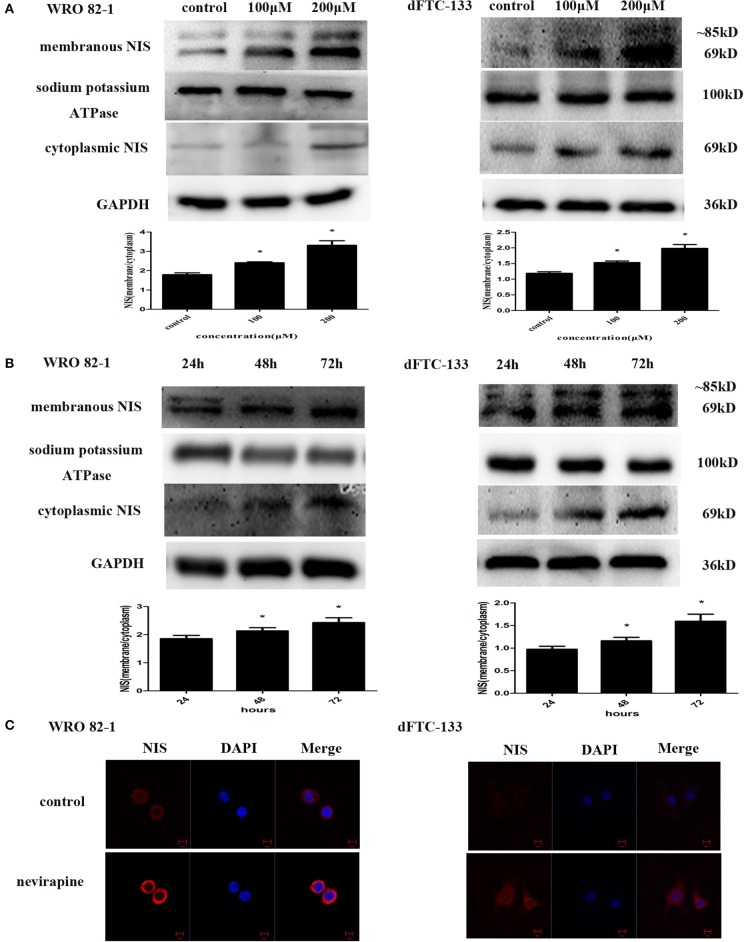
Nevirapine up-regulated cytomembrane-localized NIS protein in WRO 82-1 cells and dFTC-33 cells. **(A)** Protein expression of membranous and cytoplasmic NIS in WRO 82-1 and dFTC-133 cells treated by nevirapine for 72 h was determined by western blot analysis and analyzed quantitatively. Sodium potassium ATPase and GAPDH were used as the loading control. Data are shown as the mean ± SD of three experiments. **(B)** The expressions of NIS protein in cytomembrane and cytoplasm in both cells with 200 μM nevirapine in time course experiments were determined by western blot analysis with quantification. **(C)** Immunofluorescence of NIS protein in WRO 82-1 and dFTC-133 cells by 200 μM nevirapine for 72 h was detected by confocal microscope. Scale bar represents 10 μm. *P < 0.05 vs. control.

To determine the effect of nevirapine on NIS protein translocation, the changes in levels of membranous and cytoplasmic NIS proteins were conducted by immunoblotting with NIS-specific antibody. Treatment with nevirapine for 72 h resulted in a marked increase of plasma membrane-localized NIS protein in WRO 82-1 cells and dFTC-133 cells, with an increase of 1.8- and 1.7-fold in 200 μM nevirapine group compared with that of control group (*P* < 0.05, Figure 3A). Further results showed that the expression of plasma membrane-localized NIS protein was upregulated by nevirapine (200 μM) in a time-dependent manner, with an increase of 1.2- and 1.3-fold in WRO 82-1 and 1.2- and 1.6-fold in dFTC-133 with 200 μM nevirapine for 48 and 72 h, respectively, compared with that treated for 24 h (*P* < 0.05, Figure 3B). The localization and expression of NIS were then explored by immunofluorescence experiment. The results showed that nevirapine increased both cytoplasmic and cytomembrane-located NIS expression, the latter was more significant (Figure 3C).

### Nevirapine Increased Expression of NIS and Radioiodide Uptake by Activating PAX8 Protein in Differentiated Thyroid Cancer Cells

**Figure 4:**
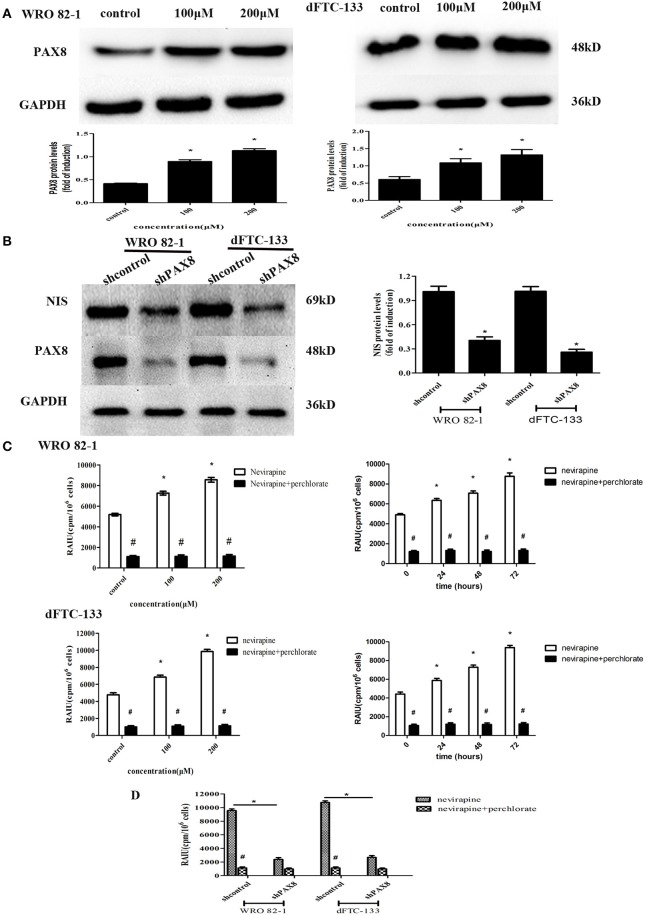
The expression of PAX8 protein was increased by nevirapine and up-regulation of NIS and radioactivity by nevirapine was related to a PAX8 dependent mechanism. **(A)** WRO 82-1 and dFTC-133 cells were treated with nevirapine for 72 h and subsequently PAX8 expression was determined by western blot analysis with quantitative analysis. GAPDH was used as the loading control. Data are shown as the mean ± SD of three experiments. **(B)** WRO 82-1 and dFTC-133 cells were transfected with sh-Control or sh-PAX8, and then treated with 200 μM of nevirapine for 72 h. The expression of NIS protein was determined by western blot analysis and analyzed quantitatively. **(C)** Radioactive iodine uptake (RAIU) in WRO 82-1 and dFTC-133 cells was determined. The sodium perchlorate was used as a selective inhibitor for NIS-mediated iodide uptake. **(D)** WRO 82-1 and dFTC-133 cells were transfected with sh-Control or sh-PAX8, and then RAIU was determined with or without sodium perchlorate. *P < 0.05 vs. control, ^#^*P* < 0.05 vs. relevant group treated by nevirapine only.

PAX8 is one of thyroid-specific transcription factors, so the expression of PAX8 protein by Western blotting was engaged to demonstrate its role in response to nevirapine. It was found that nevirapine-treated cells presented significantly increased expressions of PAX8, 2.2 times and 2.7 times in WRO 82-1 cells, 1.8 times and 2.2 times in dFTC-133 cells with 100 and 200 μM nevirapine compared with control group (all *P* < 0.05, Figure 4A). Furthermore, to verify these results, we established PAX8 knockdown cells by lentivirus. Nevirapine failed to increase the expression of NIS protein when PAX8 was knocked down (*P* < 0.05, Figure 4B). These results demonstrated that PAX8 was a key factor that mediated the function of nevirapine on NIS.

The direct effect of nevirapine on NIS-mediated radioactivity was investigated in WRO 82-1 cells and dFTC-133 cells. As expected, nevirapine-treated groups showed significantly increase in radioactive countings compared with control group in a concentration and time-dependent manner, 1.4- and 1.7-fold in WRO 82-1 cells, 1.4- and 2.1-fold in dFTC-133 cells with 100 and 200 μM nevirapine for 72 h, and 1.3-, 1.4-, and 1.8-fold in WRO 82-1 cells, 1.3-, 1.6-, and 2.1-fold in dFTC-133 cells with 200 μM nevirapine for 24, 48, and 72 h, but which were reversed by perchlorate, a sensitive NIS inhibitor (*P* < 0.05, Figure 4C). However, nevirapine-treated cells with PAX8 knockdown elicited a significant decrease in radioactivity compared with those without PAX8 knockdown (*P* < 0.05, Figure 4D). Thus, PAX8 played an important role in raising radioactivity in differentiated thyroid cancer cells by nevirapine.

### Nevirapine Increases NIS-Mediated Radioiodide Uptake by Activation of TSHR/cAMP/CREB/PAX8 Signaling Pathway in Dedifferentiated Thyroid Cancer Cells

Activation of MEK/ERK and PI3K/Akt pathways can trigger tumor spread and result in dedifferentiation (2, 16, 17). In the present study, we explored whether nevirapine increased NIS expression by inhibition of above signal pathways. The results suggested that nevirapine did not inhibit the phosphorylation of ERK1/2 and Akt, and nevirapine-induced NIS expression failed to be further significantly increased by the MEK inhibitor PD98059 and the Akt inhibitor MK2206 (all *P* > 0.05). The above results indicated that MEK/ERK and PI3K/Akt signal pathways did not play vital roles in NIS expression and radioiodide uptake induced by nevirapine or that nevirapine and inhibitors of both signal pathways acted via the same downstream pathway to induce redifferentiation of DeTC cells (Supplementary Figures 2, 3).

**Figure 5:**
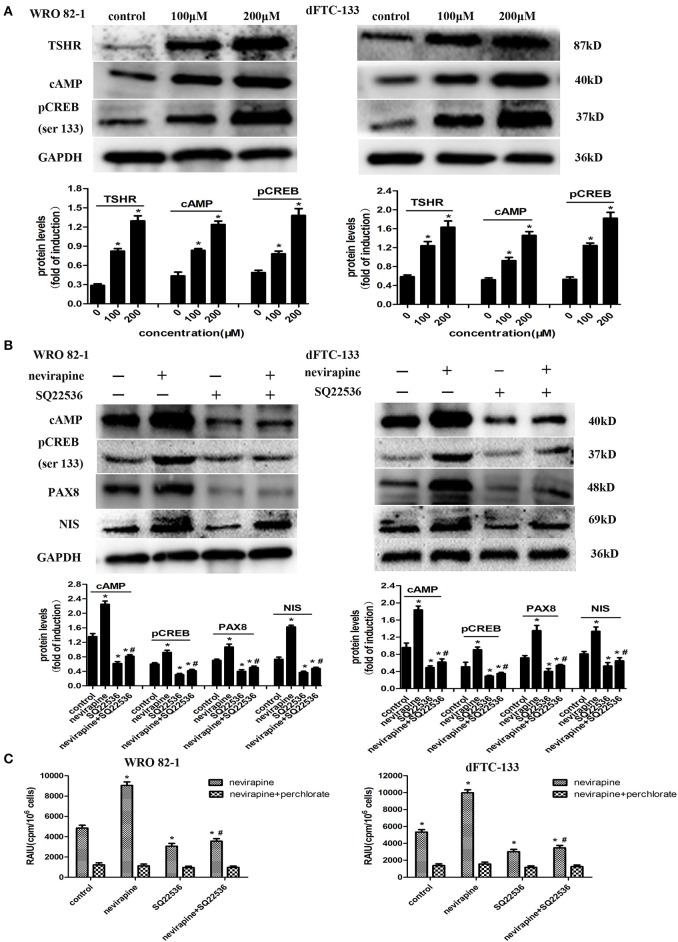
Nevirapine increased NIS-mediated radioiodide uptake by activation of TSHR/cAMP/CREB/PAX8 signaling pathway in dedifferentiated thyroid cancer cells. **(A)** The expressions of TSHR, cAMP, and pCREB (ser 133) in WRO 82-1 and dFTC133 cells after 72 h of nevirapine treatment were determined by western blot with quantitative analysis. GAPDH was used as the loading control. **(B)** The expressions of cAMP, pCREB, PAX8, and NIS in WRO 82-1 and dFTC-133 cells treated with or without 200 μM nevirapine and SQ22536 for 72 h were determined by western blot and analyzed quantitatively. **(C)** RAIU in WRO 82-1 and dFTC-133 cells was determined in treated cells with 200 μM nevirapine for 72 h with or without sodium perchlorate. The sodium perchlorate was used as a selective inhibitor for NIS-mediated iodide uptake. *P < 0.05 vs. control, ^#^*P* < 0.05 vs. relevant group treated by nevirapine only.

Numerous reports have demonstrated that activation of the TSH receptor (TSHR) is needed for optimal NIS expression and localization to plasma membrane (35, 36). We previously showed that nevirapine upregulated TSHR mRNA in anaplastic thyroid carcinoma cells, which could increase the expression of cAMP (37, 38). cAMP-response element binding protein (CREB) is a key mediator of TSH in thyroid cells, which can mediate thyroid cells function, such as, thyroid hormone production and iodide uptake (38). So, we explored whether NIS-mediated radioiodide uptake was via the activation of TSHR/cAMP/CREB/PAX8 pathway. As shown in Figure 5A, the expressions of TSHR, cAMP, and pCREB (Ser133) were upregulated significantly by nevirapine (TSHR, 3.8- and 2.8-fold, cAMP, 2.8- and 2.8-fold, pCREB (Ser133), 2.8- and 3.4-fold in WRO 82-1 and dFTC-133 cell, respectively, all *P* <0.05). Furthermore, nevirapine-induced expressions of pCREB, PAX8, NIS, and radioiodide uptake were inhibited by SQ22536, a specific cAMP inhibitor (*P* < 0.05, Figures 5B,C), which meant that the upregulation of NIS-mediated radioiodide uptake by nevirapine was dependent of TSHR/cAMP/CREB/PAX8 signal pathway.

### Nevirapine Inhibited Tumor Growth, Elevated NIS Protein Expression and Iodine Accumulation in Athymic Mouse Xenografts of WRO 82-1 Cells

**Figure 6:**
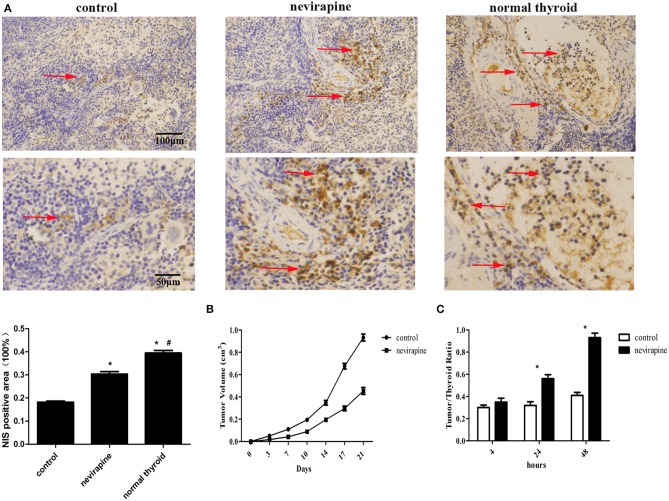
Nevirapine inhibited tumor growth and induced NIS protein expression and iodine accumulation in athymic mouse xenografts of WRO 82-1 cells. **(A)** The expression of NIS in xenografted tumors treated by nevirapine or DMSO (control) and normal human thyroid were detected by immunohistochemical staining and analyzed quantitatively. Arrowheads indicate positive staining areas. **(B)** The tumor growth in athymic mouse xenografts of WRO 82-1 cells treated by nevirapine or DMSO up to 21 days. **(C)** RAIU in nude mice treated by nevirapine or DMSO (control) was expressed as a ratio of tumor to nude mouse thyroid. Scale bar represents 100 μM (upper) and 50 μM (lower). *P < 0.05 vs. control group, ^#^*P* < 0.05 vs. nevirapine-treated group.

Ten days after inoculation of WRO 82-1 cells, the athymic mice were subjected to treatment with nevirapine and tumor sizes were determined twice a week. Three weeks after nevirapine treatment, 51% reduction in tumor growth was recorded in nevirapine-treated mice compared with that of control mice (*P* < 0.05, Figure 6B). The positive rates of NIS were 39.5 ± 1.9% in normal thyroid tissues, 30.4 ± 1.8% in xenografts of the nevirapine-treated group and 18.2 ± 0.8% in xenografts of the control group (Figure 6A). The positive rate of NIS in nevirapine-treated group was 1.7-fold compared with that in untreated group and 0.8-fold compared with that in normal thyroid tissues, which indicated that nevirapine increased the level of NIS protein significantly (*P* < 0.05), although the expression of NIS in nevirapine-treated xenografts was lower than that in normal thyroid tissues (*P* < 0.05). Consistently, nevirapine-treated xenografts exhibited a significant augment in the ratio of radioactivity between tumor and thyroid, which reached the maximum at 48 h after Na^125^I injection, 0.93 ± 0.04 (*P* < 0.05, Figure 6C), while control xenografts displayed a constant low level of radioiodide uptake.

## Discussion

The dedifferentiated thyroid carcinoma (DeTC) may occur in recurrence or metastasis of previously treated WDTC, which loses a number of markers related to thyroid cell differentiation. The degree of differentiation of thyroid cancer determines the likelihood of favorable response to radioiodine therapy, so loss of iodide uptake ability makes the tumor resistant to radioiodine therapy.

Adequate expression and membrane-targeting of NIS is a prerequisite for effective radioactive iodine therapy in thyroid cancer (24). The loss and/or abnormal trafficking (intracellular localization) of NIS in DeTC impedes therapeutic efforts, due to inability to concentrate radioiodine (6). In the present study, we assessed the therapeutic potential of nevirapine for DeTC both *in vitro* and *in vivo* by using two cell lines (WRO 82-1 and dFTC-133) and tumor-xenografted nude mice. Our results indicated that nevirapine can increase NIS-induced iodide uptake by upregulating the expression and trafficking to cell surface of NIS in DeTC cells.

PAX8, a member of the paired box (PAX) family of transcription factors expressed widely in the developing neural tube, kidney as well as adult thyroid, plays a vital role in transcription of thyroid-related genes (39–44). PAX8 upregulates the level of NIS via binding to the human upstream enhancer element (hNUE) (12, 13, 42). In line with these results, our study showed that PAX8 was significantly upregulated in response to nevirapine. When PAX8 was knockdown, nevirapine failed to increase NIS expression and radioiodide uptake, suggesting that nevirapine increased NIS-induced radioiodide uptake via activation of PAX8.

Molecular abnormalities are believed to be involved in radioiodine-refractory thyroid cancer, such as, activating mutations in BRAF, RAS and PTEN, RET-PTC rearrangements, PPARγ-PAX8 rearrangements, mutations of PI3KCA and AKT1, and most of them have been noted in genes related to MEK/ERK and PI3K/Akt pathway (16, 17). The inhibitors of these signaling pathways are used to improve radioiodine resistance in iodine-refractory DTC, although long-term prognosis clinically is not satisfactory (18). Previous study revealed that the WRO 82-1 cell line expressed mutant BRAF V600E (45), which could activate MEK/ERK signal pathway, and FTC-133 cell line had hemizygous deletion of PTEN (46), which could further activate PI3K/Akt signal pathway. Therefore, we explored if NIS mediated radioiodide uptake via the two pathways. Regrettably, the results indicated that inhibition of MEK/ERK and PI3K/Akt signal pathway played a very slender role in expression of NIS and radioiodide uptake induced by nevirapine or that nevirapine and inhibitors of both signal pathways acted through the same downstream pathway to induce redifferentiation of thyroid cancer cells.

Dedifferentiation of thyroid cancer may lead to the decreased expression of TSHR and diminished signal transduction after TSHR activation (24, 25). Our previous study showed that the expression of TSHR was enhanced by nevirapine in anaplastic thyroid carcinoma cells (25). Similar result was found in the present study. TSHR, a G protein–coupled receptor, potentially couples to all G protein families, and mainly activates Gs/adenylate cyclase signaling pathway. Activation of the Gs pathway stimulates the production of intracellular cAMP and phosphorylation of cAMP response element-binding protein (CREB), which regulate of thyrocyte growth, tumorigenesis, cancer progression, and differentiation (47–49). However, few literatures reported the relationship between TSHR/cAMP/CREB pathway and radioactive iodine. It is unclear whether this pathway is involved in re-differentiation, especially increased radioiodide uptake of DeTC by nevirapine. The current study showed that nevirapine increased the expressions of cAMP and pCREB (Ser133), and the results were reversed when a cAMP inhibitor, SQ22536, was administered. Moreover, nevirapine-induced up-regulation of PAX8, NIS, and radioiodide uptake were significantly inhibited by SQ22536. These results demonstrated that nevirapine induced upregulation of NIS and radioiodide uptake via TSHR/cAMP/CREB/PAX8 signal pathway in DeTC.

There are limitations in our study. The primary cells are more similar to the initial thyroid cancer cells than cell lines. Antonelli et al. studied the effect of new pyrazolo [3,4-d] pyrimidine compounds on dedifferentiated papillary thyroid cancer and evaluated the chemosensitivity of anaplastic thyroid cancer with primary cells (50, 51). So, we will conduct further research in primary dedifferentiated thyroid cancer cells from DeTC patients. Besides, the effect of nevirapine on NIS-induced radioiodide uptake in patients with DeTC was not performed in our study. The related clinical studies were rather limited so far. However, there was a case report. Modoni et al. described a successful case of 76-year-old woman with a follicular variant of a papillary thyroid carcinoma progressed to DeTC (13). Two months after nevirapine treatment, metastatic lesions exhibited significant increase in radioiodine uptake, most of the bone metastasis disappeared and no new metastasis was revealed during the whole nevirapine treatment. More clinical trials are needed before nevirapine could be administrated in patients with DeTC.

In summary, our present findings showed that nevirapine could restore iodine uptake in dedifferentiated thyroid cancer by up-regulation of NIS expression and translocation to cell membrane *in vitro* and *in vivo*, which provided some valuable evidence that nevirapine might be a promising anticancer drug in treatment of dedifferentiated thyroid cancers.

## Data Availability Statement

All datasets generated for this study are included in the article/Supplementary Material.

## Ethics Statement

The animal study was reviewed and approved by the ethical committee of Shandong Provincial Qianfoshan Hospital, Shandong University.

## Author Contributions

LL, JD, and HS designed the project. HS and JZ performed the mice and cell experiments. HS wrote the main manuscripts. HS and JY analyzed and interpreted data. All authors reviewed the manuscript.

### Conflict of Interest

The authors declare that the research was conducted in the absence of any commercial or financial relationships that could be construed as a potential conflict of interest.

## Supplementary Material

Supplementary Figure 1 The dFTC-133 cells were established by monoclonal culturing after 15μCi^131^I radiation for 3 days. **(A)** Relative mRNA expressions of thyroid specific makers were calculated by RT-PCR. **(B)** NIS protein in parent and clone cells was detected by immunofluorescence assay. **(C)** RAIU in both cells was determined and dFTC-133 had stable lower RAIU than FTC-133. GAPDH was used as the loading control. ^*^*P* < 0.05 vs. control.

Supplementary Figure 2 Nevirapine-induced NIS expression was not mediated by inhibition of MEK/ERK pathway. WRO 82-1 and dFTC-133 cells were treated with 200 μM nevirapine for 72 h in the presence or absence of 10 μM PD98059, an MEK inhibitor. Western blot analysis shows the protein expressions of pERK1/2 and NIS. GAPDH was used as the loading control. ^*^*P* < 0.05 vs. control.

Supplementary Figure 3 Nevirapine-induced NIS expression was not mediated by inhibition of PI3K/Akt pathway. WRO 82-1 and dFTC-133 cells were treated with 200 μM nevirapine for 72 h in the presence or absence of 10 μM MK2206, an Akt inhibitor. Western blot analysis shows the protein expressions of pAkt and NIS. GAPDH was used as the loading control. ^*^*P* < 0.05 vs. control.
