# Supplementary figures and images for: Nevirapine Increases Sodium/Iodide Symporter-Mediated Radioiodide Uptake by Activation of TSHR/cAMP/CREB/PAX8 Signaling Pathway in Dedifferentiated Thyroid Cancer
Source: Front Oncol. 2020 Mar 31;10:404. doi: 10.3389/fonc.2020.00404 (PMC7145398; doi:10.3389/fonc.2020.00404)

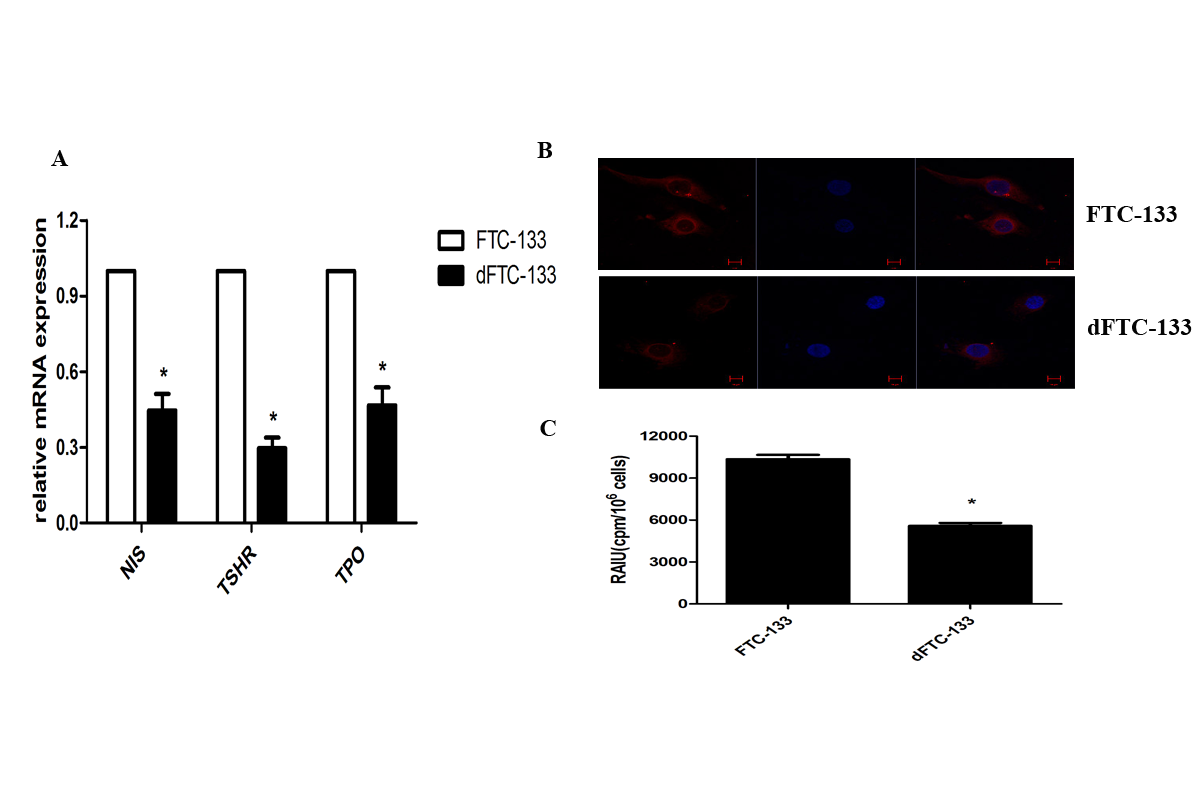

Supplement: Supplementary Figure 1 — The dFTC-133 cells were established by monoclonal culturing after 15μCi131I radiation for 3 days. (A) Relative mRNA expressions of thyroid specific makers were calculated by RT-PCR. (B) NIS protein in parent and clone cells was detected by immunofluorescence assay. (C) RAIU in both cells was determined and dFTC-133 had stable lower RAIU than FTC-133. GAPDH was used as the loading control. *P < 0.05 vs. control. [file Image_1.TIF]

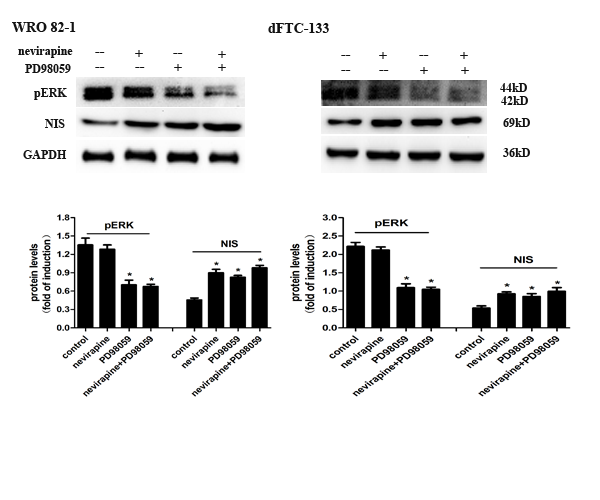

Supplement: Supplementary Figure 2 — Nevirapine-induced NIS expression was not mediated by inhibition of MEK/ERK pathway. WRO 82-1 and dFTC-133 cells were treated with 200 μM nevirapine for 72 h in the presence or absence of 10 μM PD98059, an MEK inhibitor. Western blot analysis shows the protein expressions of pERK1/2 and NIS. GAPDH was used as the loading control. *P < 0.05 vs. control. [file Image_2.TIF]

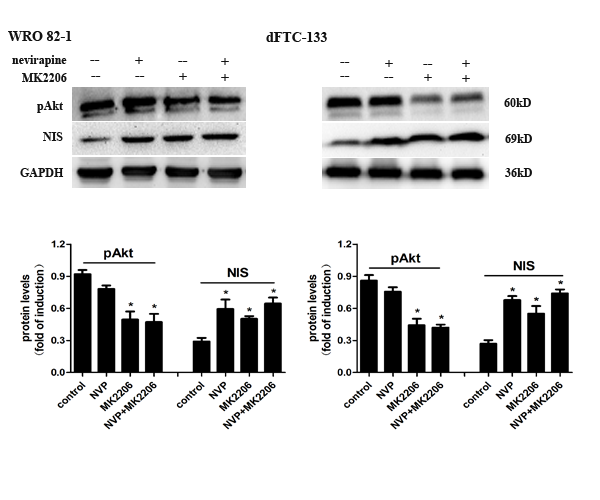

Supplement: Supplementary Figure 3 — Nevirapine-induced NIS expression was not mediated by inhibition of PI3K/Akt pathway. WRO 82-1 and dFTC-133 cells were treated with 200 μM nevirapine for 72 h in the presence or absence of 10 μM MK2206, an Akt inhibitor. Western blot analysis shows the protein expressions of pAkt and NIS. GAPDH was used as the loading control. *P < 0.05 vs. control. [file Image_3.TIF]
